# Supplementary material for: Coffee Beverage: A New Strategy for the Synthesis of Polymethacrylates via ATRP
Source: Molecules. 2022 Jan 27;27(3):840. doi: 10.3390/molecules27030840 (PMC8840111; doi:10.3390/molecules27030840)
Supplement: Supplementary file 1 [file molecules-27-00840-s001.zip › molecules-1529687-supplementary.pdf]

Supporting data for

Coffee beverage: A new strategy for the synthesis of polymethacrylates *via* ATRP

Karolina Surmacz,[a] Paweł Błoniarz,[b] Paweł Chmielarz,[b,*]

[a] Doctoral School of Engineering and Technical Sciences, Rzeszow University of Technology, Al. Powstańców Warszawy 8, 35-959 Rzeszów, Poland

[b] Department of Physical Chemistry, Faculty of Chemistry, Rzeszow University of Technology, Al. Powstańców Warszawy 6, 35-959 Rzeszów, Poland

[*] Corresponding author. E-mail: p_chmiel@prz.edu.pl

Table of contents

S1. Synthesis procedures 4

S1.1 General procedure for the synthesis of PDMAEMA by ARGET ATRP in pure water 4

S1.2 General procedure for the synthesis of PDMAEMA by ARGET ATRP in coffee solution 4

S1.3 The procedure for the synthesis of PGMA by ARGET ATRP in coffee solution 4

S1.4 The procedure for the synthesis of POEGMA by ARGET ATRP in coffee solution 4

S1.5 The procedure for the synthesis of PBA by ARGET ATRP in miniemulsion coffee solution 5

S2. ARGET ATRP of DMAEMA in various temperature 6

S2.1 Kinetic investigation 6

S2.2 GPC analysis 6

S3. ARGET ATRP in various NaBr concentration 7

S3.1 Kinetic investigation 7

S4. ARGET ATRP of DMAEMA in various coffee beans extract 8

S4.1 GPC analysis 8

S4.2 ^{1}H NMR analysis of coffee extract 9

S4.2.1 ^{1}H NMR analysis of Segafredo Arabica and Segafredo Espresso CASA extracts 9

S5. ARGET ATRP in various concentration of coffee extract 10

S5.1 GPC analysis 10

S6. ARGET ATRP of various monomers 10

S6.1 GPC analysis 10

S6.2 ARGET ATRP of n-BA in miniemulsion media 11

S6.3 ^{1}H NMR analysis of polymers 12

S6.3.1 ^{1}H NMR analysis of PDMAEMA 12

S6.3.2 ^{1}H NMR analysis of PGMA 14

S6.3.3 ^{1}H NMR analysis of POEGMA 14

S6.3.4 ^{1}H NMR analysis of P*n*BA 15

S7. Chain-extension of PDMAEMA-Br macroinitiator 15

S8. DPV analysis 17

S9. HPLC analysis 18

S10. ARGET ATRP of DMAEMA in 7.0 mM caffeine solution 18

S1. Synthesis procedures

S1.1 General procedure for the synthesis of PDMAEMA by ARGET ATRP in pure water

Sodium bromide (NaBr) (82.3 mg, 0.8 mmol) was dissolved in distilled water (4.0 mL) into 10 mL Schlenk flask equipped with a magnetic stirring bar. A stock solution of DMAEMA (3.2 mL, 19 mmol) and 139 μL of 10% solution of ethyl 2-bromoisobutyrate (EBiB) in DMF were added. The Schlenk flask was closed with a rubber septum and solution degassed with argon for 20 minutes. Thereafter, the reaction was started while the $Cu^{II}Br_2$/TPMA stock solution (0.05 M in water) was injected (23.8 μL). The synthesis was conducted with a vigorous stirring rate (800 rpm) at room temperature. Samples of the reaction mixture were withdrawn periodically to follow the monomer conversion using ^{1}H NMR. The M_n and MWD were determined by GPC measurements using DMF + 10 mM LiCl as eluent.

S1.2 General procedure for the synthesis of PDMAEMA by ARGET ATRP in coffee solution

Sodium bromide (NaBr) (82.3 mg, 0.8 mmol) was dissolved in water coffee extract (4.0 mL) into 10 mL Schlenk flask equipped with a magnetic stirring bar. A stock solution of DMAEMA (3.2 mL, 19 mmol) and 139 μL of 10% solution of ethyl 2-bromoisobutyrate (EBiB) in DMF were added. The Schlenk flask was closed with a rubber septum and solution degassed with argon for 20 minutes. Thereafter, the reaction was started while the $Cu^{II}Br_2$/TPMA stock solution (0.05 M in water) was injected (23.8 μL). The synthesis was conducted with a vigorous stirring rate (800 rpm) at room temperature. Samples of the reaction mixture were withdrawn periodically to follow the monomer conversion using ^{1}H NMR. The M_n and MWD were determined by GPC measurements using DMF + 10 mM LiCl as eluent.

S1.3 The procedure for the synthesis of PGMA by ARGET ATRP in coffee solution

Sodium bromide (82.3 mg, 0.8 mmol) was dissolved in water coffee extract (4.0 mL) into 10 mL Schlenk flask equipped with a magnetic stirring bar. A stock solution of GMA (3.2 mL, 24 mmol) and 178 μL of 10% solution of EBiB in DMF were added. The Schlenk flask was closed with a rubber septum and solution degassed with argon for 20 minutes. Thereafter, the reaction was started while the $Cu^{II}Br_2$/TPMA stock solution (0.05 M in water) was injected (25.2 μL, 1.19 μmol). The synthesis was conducted with a vigorous stirring rate (800 rpm) at room temperature. Samples of the reaction mixture were withdrawn periodically to follow the monomer conversion using ^{1}H NMR. The M_n and MWD were determined by GPC measurements using DMF + 10 mM LiCl as eluent.

S1.4 The procedure for the synthesis of POEGMA by ARGET ATRP in coffee solution

Sodium bromide (82.3 mg, 0.8 mmol) was dissolved in water coffee extract (4.0 mL) into 10 mL Schlenk flask equipped with a magnetic stirring bar. A stock solution of OEGMA (3.2 mL, 7 mmol) and 51 μL of 10% solution of EBiB in DMF were added. The Schlenk flask was closed with a rubber septum and solution degassed with argon for 20 minutes. Thereafter, the reaction was started while the $Cu^{II}Br_2$/TPMA stock solution (0.05 M in water) was injected (25 μL, 1.25 μmol). The synthesis was conducted with a vigorous stirring rate (800 rpm) at room temperature. Samples of the reaction mixture were withdrawn periodically to follow the monomer conversion using ^{1}H NMR. The M_n and MWD were determined by GPC measurements using DMF + 10 mM LiCl as eluent.

S1.5 The procedure for the synthesis of PBA by ARGET ATRP in miniemulsion coffee solution

A stock solution of *n*-butyl acrylate (*n*BA) (2.02 mL, 14.0 mmol), EBiB (71.2 μL, 0.14 mmol) and n-hexadecane (HD) (0.21 mL, 0.73 mmol) was prepared in the first baker. In the second baker was prepared 200 μL $Cu^{II}Br_2$/TPMA solution (0.05 M in water), NaBr (105 mg, 1.02 mmol), and SDS (112 mg, 0.39 mmol) were dissolved in 9.5 mL of coffee solution. The organic and aqueous solutions were mixed and ultrasonicated (130 W, 20 kHz) using sonificator (VCX 130, Sonics) by 20 minutes. Followed mixture was added to Schlenk flask and purged by argon for 20 minutes. The reaction was conducted in a vigorous stirring rate (950 rpm) at room temperature. Samples were withdrawn periodically to follow the monomer conversion by gravimetric analysis, while M_n and $Đ$ were determined by GPC measurements using THF as eluent.

S2. ARGET ATRP of DMAEMA in various temperature

S2.1 Kinetic investigation

Table S1. ARGET ATRP of DMAEMA in various temperature

Entry[1]	T (°C)	t (h)	Conv[2] (%)	DP_{app}[3]	k_p^{app}[2] (h^{-1})	$M_{n,th}$[3] (×10^{-3})	$M_{n,app}$[4] (×10^{-3})	$Đ$[4]
1	10	1.17	35.3	71	0.37	11.3	99.9	1.81
2	RT	0.5	59.4	119	1.79	19.0	71.3	1.63

[1]General reaction conditions: $[DMAEMA]_0/[EBiB]_0/[Cu^{II}Br_2]_0/[TPMA]_0$: 200/1/0.01/0.02; V_{tot} = 8 mL (in DMAEMA/ 10% coffee extract = 0.4/0.6 by v/v), $[DMAEMA]_0$ = 24 mM, $[I]_0$ = 0.12 mM. $[Cu^{II}Br_2]_0$ = 1.19 µM (2-fold excess of TPMA), [NaBr] = 0.1 M, RT = 22°C

[2] Monomer conversion, polymerization rate (k_p^{app}) and DP_{app} were determined by ^{1}H NMR.

[3] $M_{n,th} = ([M]_0/[I]_0) \times \text{Conv} \times M_{monomer} + M_{initiator}$.

[4] $M_{n,app}$ and $Đ$ was determined by DMF + 10 mM LiCl GPC with PS standards.

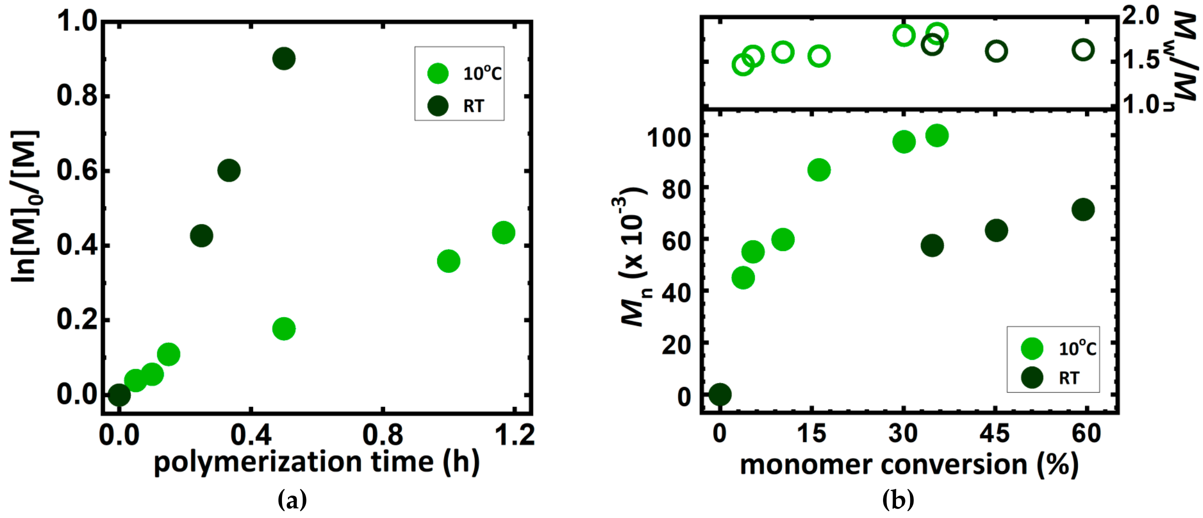

Figure S1. (a) Semilogarithmic kinetic plot, (b) M_n and M_w/M_n *vs.* monomer conversion for preparation of PDMAEMA in coffee solution at various temperature (Table S1, entries 1-2). Reaction conditions as in Table S1.

S2.2 GPC analysis

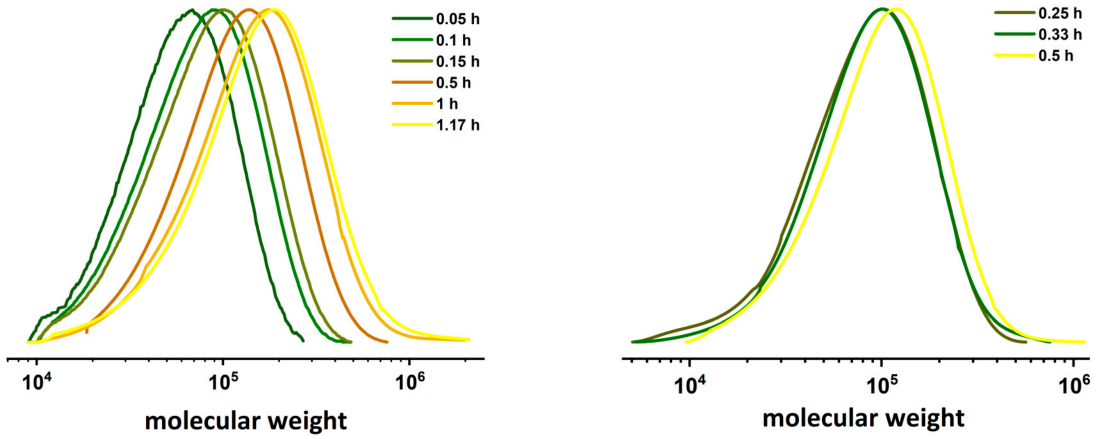

(a) (b)

Figure S2. GPC traces of PDMAEMA prepared according to (a) Table S1 entry 1 and (b) Table S1 entry 2 using as eluent DMF + 10 mM LiCl.

S3. ARGET ATRP in various NaBr concentration

S3.1 Kinetic investigation

Table S2. ARGET ATRP in various NaBr concentration in 7.5% solution received from equally proportional blend of Arabica and Robusta beans.

Entry[1]	NaBr conc.[2] (M)	Conv[3] (%)	DP_{app}[3]	k_p^{app}[3] (h^{-1})	$M_{n,th}$[4] (×10^{-3})	$M_{n,app}$[5] (×10^{-3})	$Đ$[5]	f[6] (%)
1	0.0	29.5	59	0.70	9.5	78.0	2.04	819
2	0.1	70.9	142	2.55	22.6	62.8	1.63	278
3	0.3	58.0	116	1.71	18.5	41.3	1.92	223

[1] General reaction conditions: $[DMAEMA]_0/[EBiB]_0/[Cu^{II}Br_2]_0/[TPMA]_0$: 200/1/0.01/0.02; T = 22°C; V_{tot} = 8 mL (DMAEMA/solvent = 0.4/0.6 by v/v), $[DMAEMA]_0$ = 19 mM, $[I]_0$ = 0.09 mM. $[Cu^{II}Br_2]_0$ = 1.19 μM (2-fold excess of TPMA), t = 0.5 h. Solvent: 7.5% solution of Arabica & Robusta (50/50%).

[2] Concentration with respect to the total volume.

[3] Monomer conversion, polymerization rate (k_p^{app}) and DP_{app} were determined by ^{1}H NMR.

[4] $M_{n,th} = ([M]_0/[I]_0) \times Conv \times M_{monomer} + M_{initiator}$.

[5] $M_{n,app}$ and $Đ$ was determined by DMF + 10 mM LiCl GPC with PS standards.

[6] Initiation efficiency calculated as: $f = M_{n,app}/M_{n,th}$.

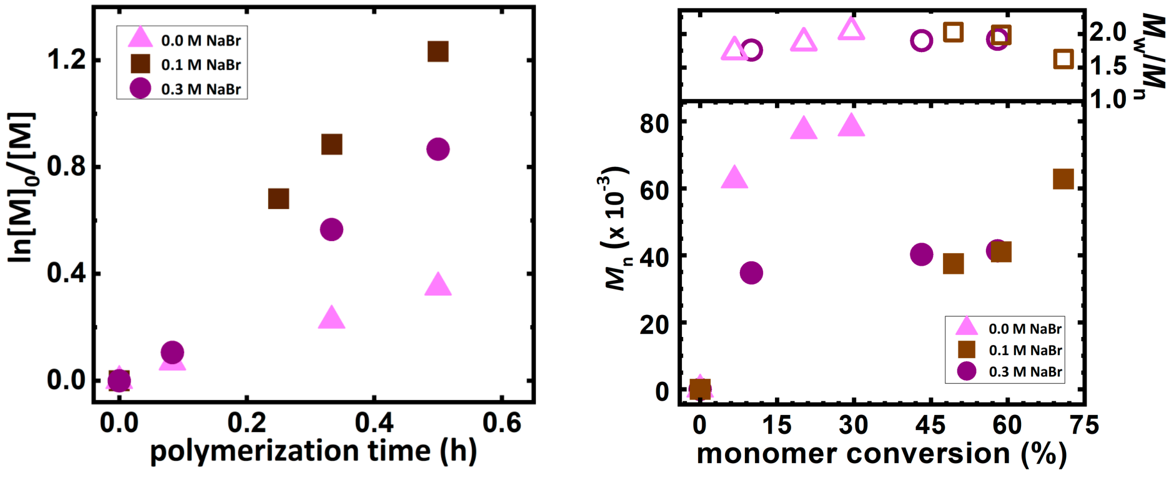

(a) (b)

Figure S3. (a) Semilogarithmic kinetic plot, (b) M_n and M_w/M_n *vs.* monomer conversion for ARGET ATRP in preparation of PDMAEMA in various NaBr concentration (Table S2). Reactions conditions as in Table S2.

S4. ARGET ATRP of DMAEMA in various coffee beans extract

S4.1 GPC analysis

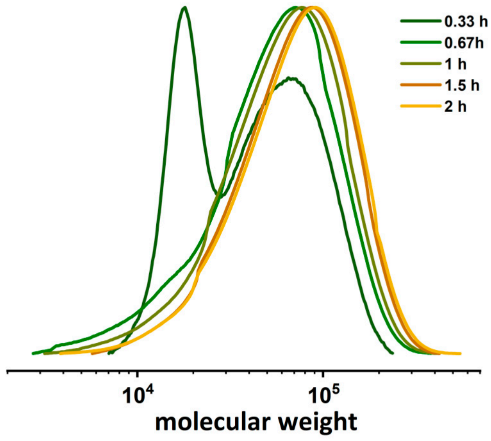

(a)

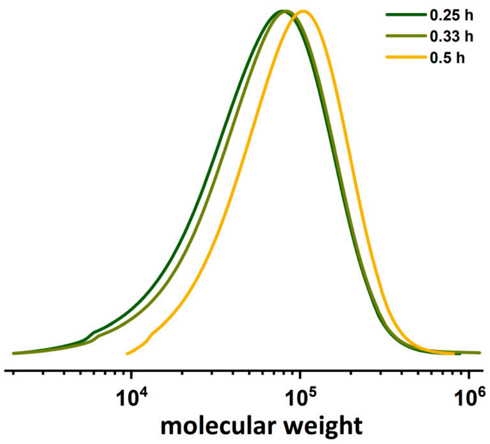

(b)

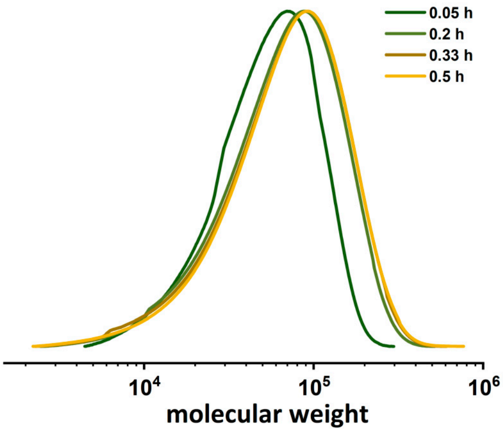

(c)

Figure S4. GPC traces of PDMAEMA prepared according to (a) Table 1 entry 1; (b) Table 1 entry 2 and (c) Table 1 entry 3 using as eluent DMF + 10 mM LiCl.

S4.2 ^{1}H NMR analysis of coffee extract

S4.2.1 ^{1}H NMR analysis of Segafredo Arabica and Segafredo Espresso CASA extracts

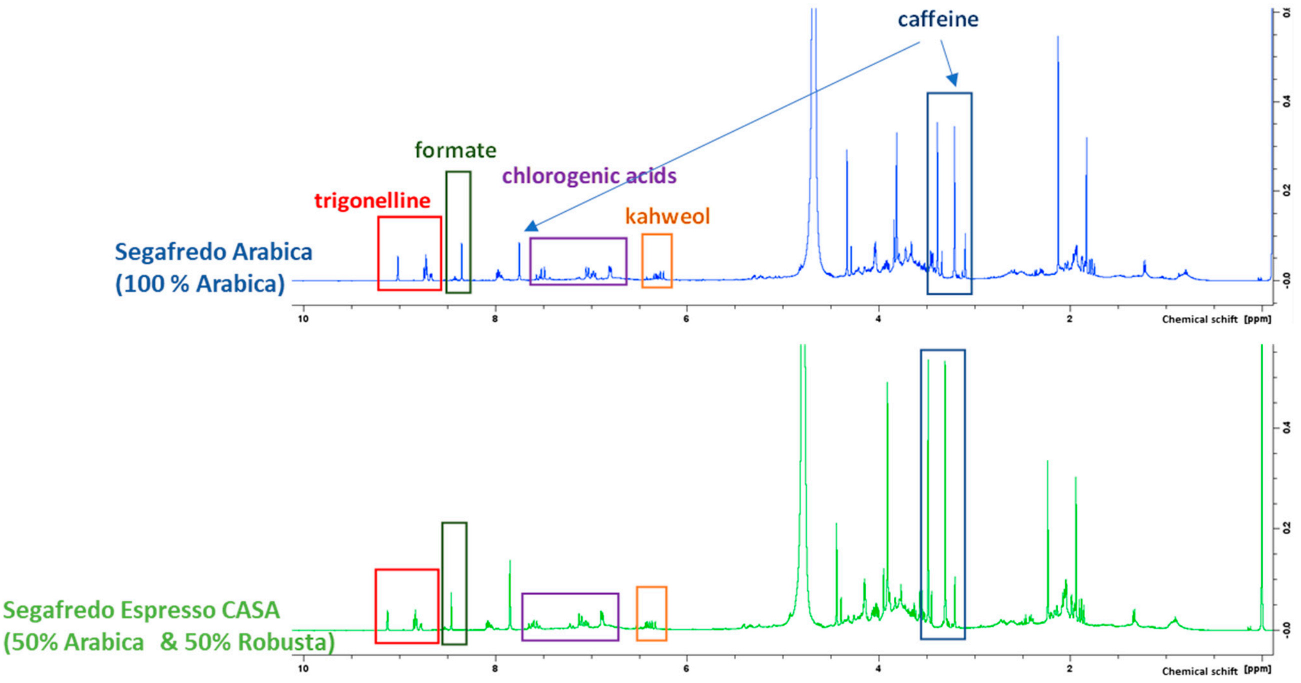

Figure S5. ^{1}H NMR stack plot of coffee samples: Segafredo Arabica and Segafredo Espresso CASA in D_2O (500 MHz, 25°C) [1, 2].

S5. ARGET ATRP in various concentration of coffee extract

S5.1 GPC analysis

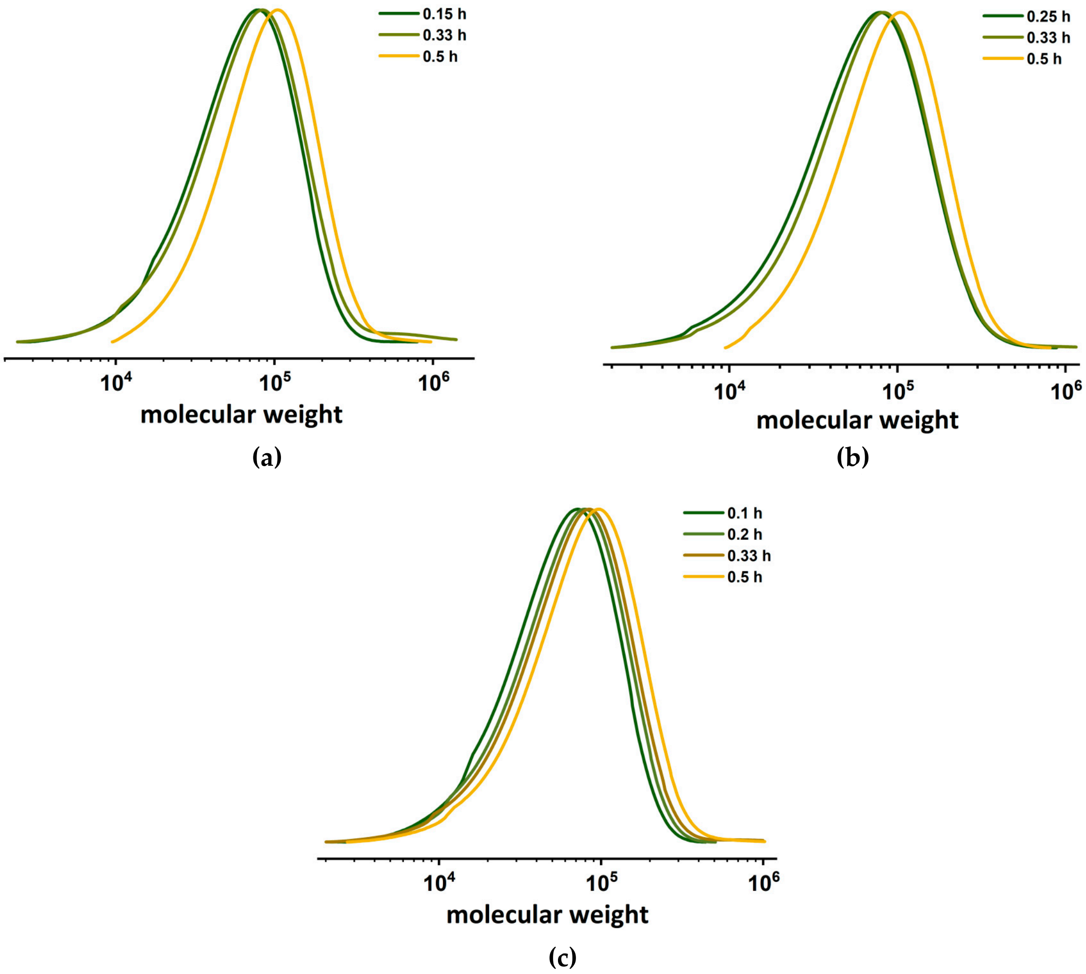

Figure S6. GPC traces of PDMAEMA prepared according to (a)Table 2 entry 1; (b) Table 2 entry 2 and (c) Table 2 entry 3 using as eluent DMF + 10 mM LiCl.

S6. ARGET ATRP of various monomers

S6.1 GPC analysis

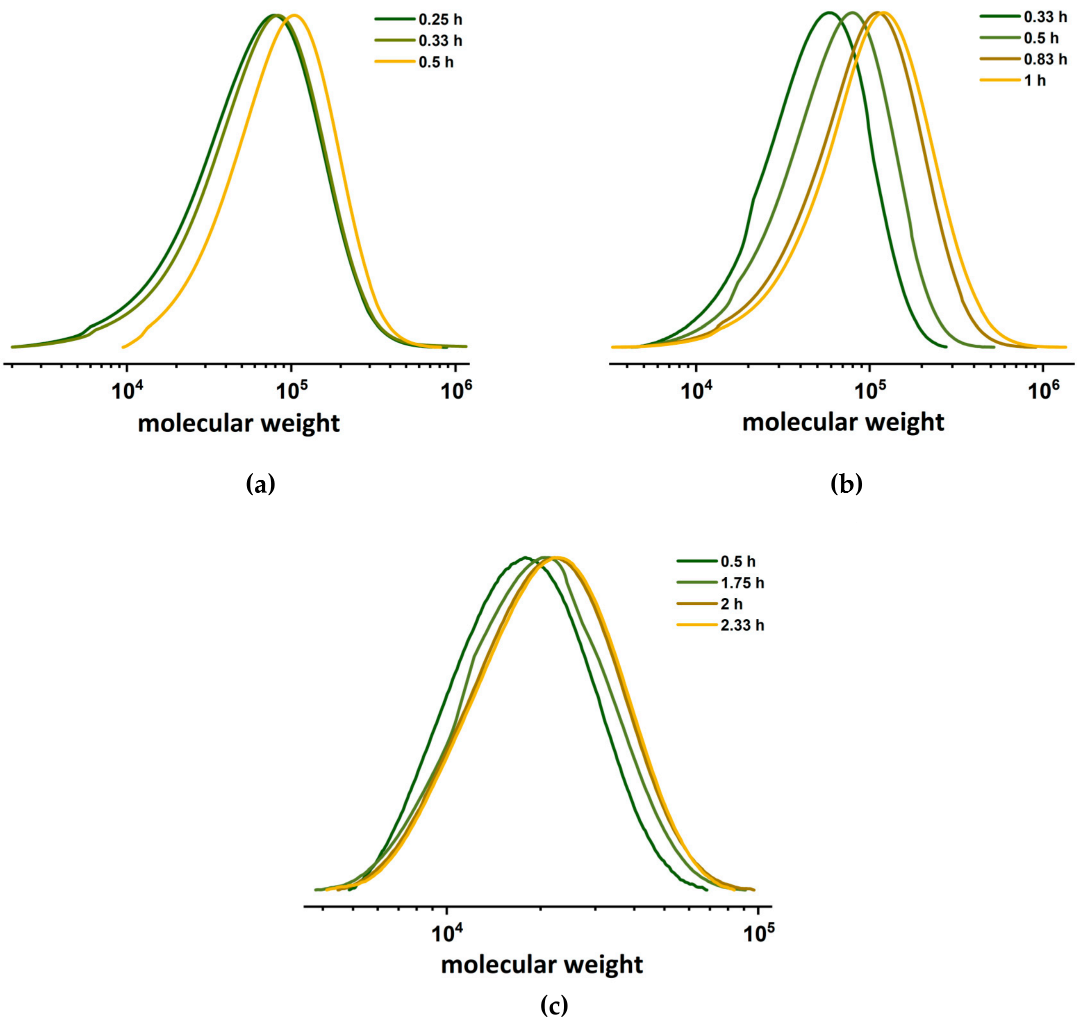

Figure S7. GPC traces of (a) PDMAEMA; (b) PGMA; (c) POEGMA chains prepared according to (a) Table 3 entry 1; (b) Table 3 entry 2 and (c) Table 3 entry 3 using as eluent DMF + 10 mM LiCl.

S6.2 ARGET ATRP of nBA in miniemulsion media

Table S3. ARGET ATRP of *n*BA in miniemulsion medium of 10% Arabica & Robusta (1:1) coffee solution[a]

Entry[1]	t (h)	Conv[2] (%)	DP_{app}[3]	k_p^{app} [2] (h^{-1})	$M_{n,th}$[3] ($\times 10^{-3}$)	$M_{n,app}$[4] ($\times 10^{-3}$)	$Đ$ [4]
1	18	80.8	81	0.093	10.5	33.8	3.07

[1]General reaction conditions: monomer 18.3 vol % in 10% coffee extract; T = 22°C; V_{tot} = 12 mL, $[Cu^{II}Br_2/TPMA]$ = 0.82 mM, [SDS] = 3.25 mM, [HD] = 70.8 mM, [NaBr] = 85 mM.

[2] Monomer conversion, polymerization rate (k_p^{app}) and DP_{app} were determined by ^{1}H NMR.

[3] $M_{n,th} = ([M]_0/[I]_0) \times \text{Conv} \times M_{monomer} + M_{initiator}$.

[4] $M_{n,app}$ and $Đ$ was determined by THF GPC with PS standards.

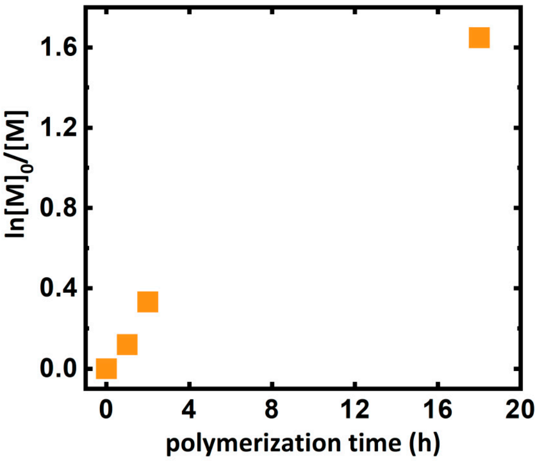

(a)

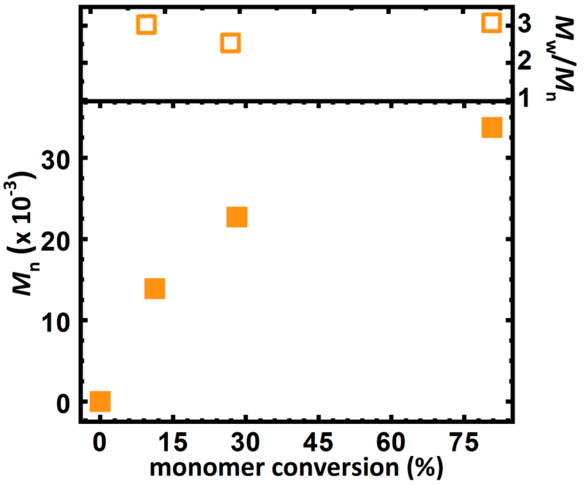

(b)

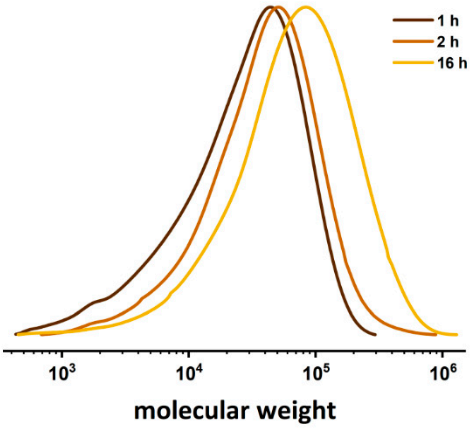

(c)

Figure S8. Polymerization of *n*BA in miniemulsion system: (a) First-order kinetic plot of monomer conversion *vs.* polymerization time. (b) M_n and M_w/M_n *vs.* monomer conversion. (c) GPC traces of P*n*BA with THF as the eluent.

S6.3 ¹H NMR analysis of polymers

S6.3.1 ¹H NMR analysis of PDMAEMA

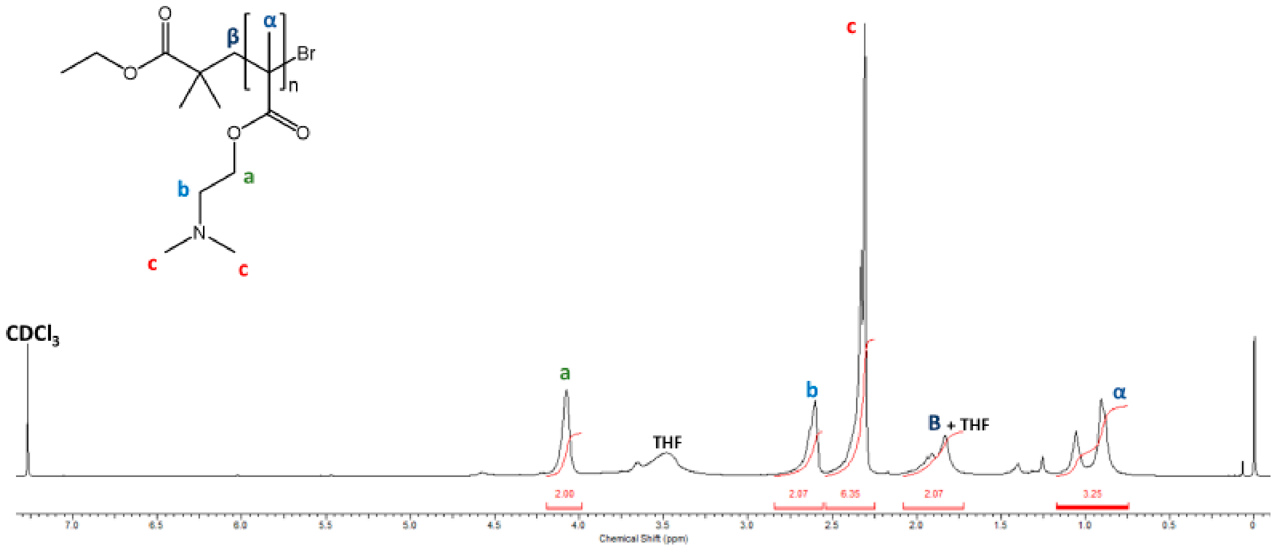

Figure S9. ^{1}H NMR spectrum of PDMAEMA homopolymer (after purification) in $CDCl_3$ (500 MHz, 25°C). The chemical shifts characteristic for PDMAEMA chains were assigned: δ (ppm) = 0.72-1.15 (3H, –CH_3, α), 1.72-2.07 (2H, –CH_2–, β), 2.23-2.53 (6H, 2 x -CH_3, c), 2.57-2.84 (2H, –CH_2–, b) and 3.99-4.19 (2H, –CH_2–, a).

S6.3.2 ¹H NMR analysis of PGMA

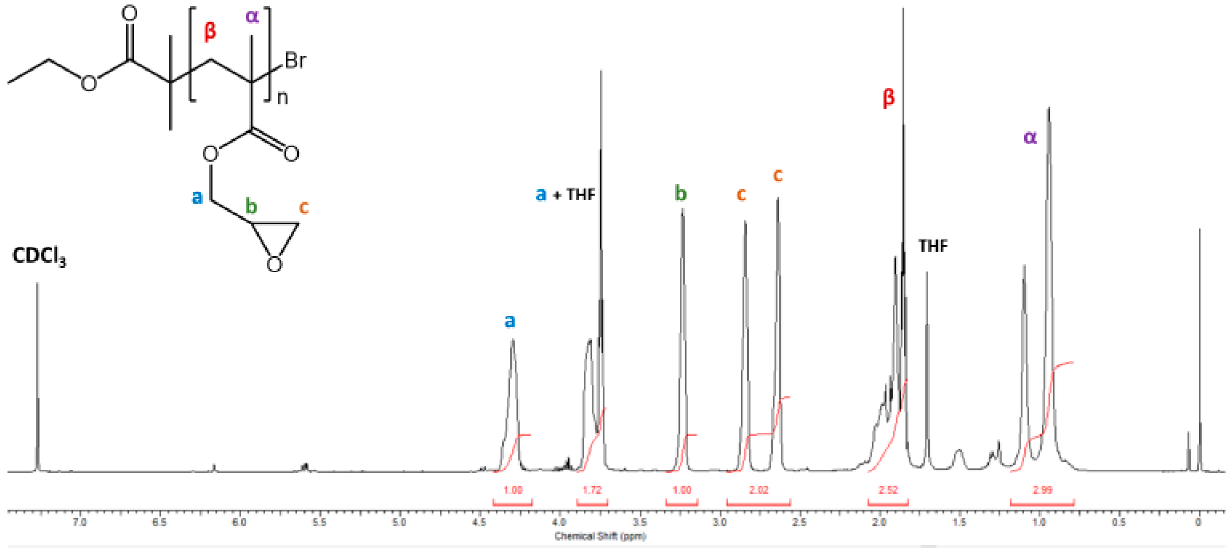

Figure S9. ¹H NMR spectrum of PGMA homopolymer (after purification) in $CDCl_3$ (500 MHz, 25°C). The chemical shifts characteristic for PGMA chains were assigned: δ (ppm) = 0.80-1.18 (3H, –CH_3, α), 1.84-2.06 (2H, –CH_2–, β), 2.56-2.96 (2H, –CH_2–, c), 3.15-3.34 (1H, –CH–, b), 3.71-3.89 and 4.19-4.39 (2H, –CH_2–, a).

S6.3.3 ¹H NMR analysis of POEGMA

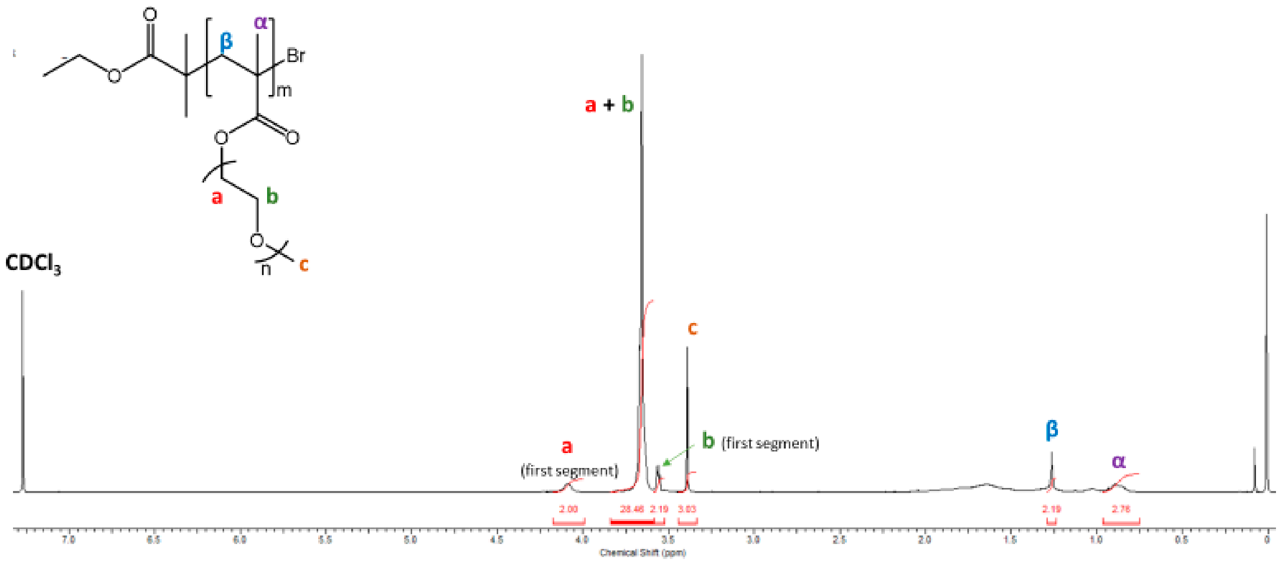

Figure S11. ¹H NMR spectrum of POEGMA homopolymer (after purification) in $CDCl_3$ (500 MHz, 25°C). The chemical shifts characteristic for POEGMA chains were assigned: δ (ppm) = 0.78-0.97 (3H, –CH_3, α), 1.23-1.30 (2H, –CH_2–, β), 3.34-3.44 (3H, CH_3–, c), 3.54-3.58 (2H, –CH_2–, b-first segment), 3.59-3.82 (28H, –CH_2– and –CH_2–, a + b), and 4.01-4.17 (2H, –CH_2–, a-first segment).

S6.3.4 ^{1}H NMR analysis of P*n*BA

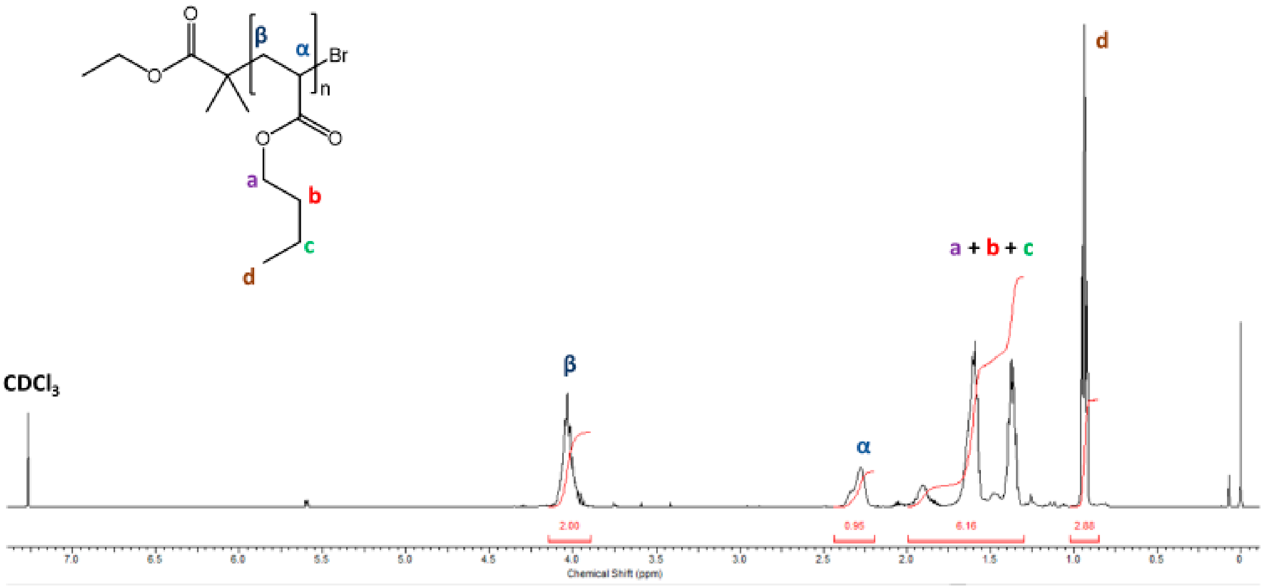

Figure S12. ^{1}H NMR spectrum of P*n*BA homopolymer (after purification) in $CDCl_3$ (500 MHz, 25°C). The chemical shifts characteristic for PBA chains were assigned: δ (ppm) = 0.85-1.01 (3H, –CH_3, d), 1.31-2.00 (6H, –CH_2–, a + b + c), 2.20-2.43 (1H, –*CH*=, *α*) and 3.90-4.14 (2H, –CH_2–, β).

S7. Chain-extension of PDMAEMA-Br macroinitiator

Table S4. Chain-extension reaction of PDMAEMA-Br in 7.5% coffee solution

Entry[1]	t (h)	Conv[2] (%)	DP_{app}[3]	k_p^{app} [2] (h^{-1})	$M_{n,th}$[3] (×10^{-3})	$M_{n,app}$[4] (×10^{-3})	*Đ* [4]
First block	0.25	25.3	38	1.18	6.3	43.3	1.70
Second block	0.17	13.3	20	0.91	13.1	62.0	1.90

[1]General reaction conditions: $[DMAEMA]_0/[EBiB]_0/[Cu^{II}Br_2]_0/[TPMA]_0$: 150/1/0.01/0.02 (first segment); $[DMAEMA]_0/[PDMAEMA\text{-}Br]_0$: 3.9/1 (second segment); T = 22°C; V_{tot} = 8 mL (DMAEMA/ 7.5% coffee Arabica & Robusta (50/50%) extract = 0.4/0.6 by v/v); $[DMAEMA]_0$ = 19 mM, $[I]_0$ = 0.13 mM. $[Cu^{II}Br_2]$= 1.19 µM (in order to stabilize copper complex applied 2-fold excess of TPMA), [NaBr] = 0.1 M.

[2] Monomer conversion, polymerization rate (k_p^{app}) and DP_{app} were determined by ^{1}H NMR.

[3] $M_{n,th} = ([M]_0/[I]_0) \times Conv \times M_{monomer} + M_{initiator}$.

[4] $M_{n,app}$ and *Đ* was determined by DMF + 10 mM LiCl GPC with PS standards.

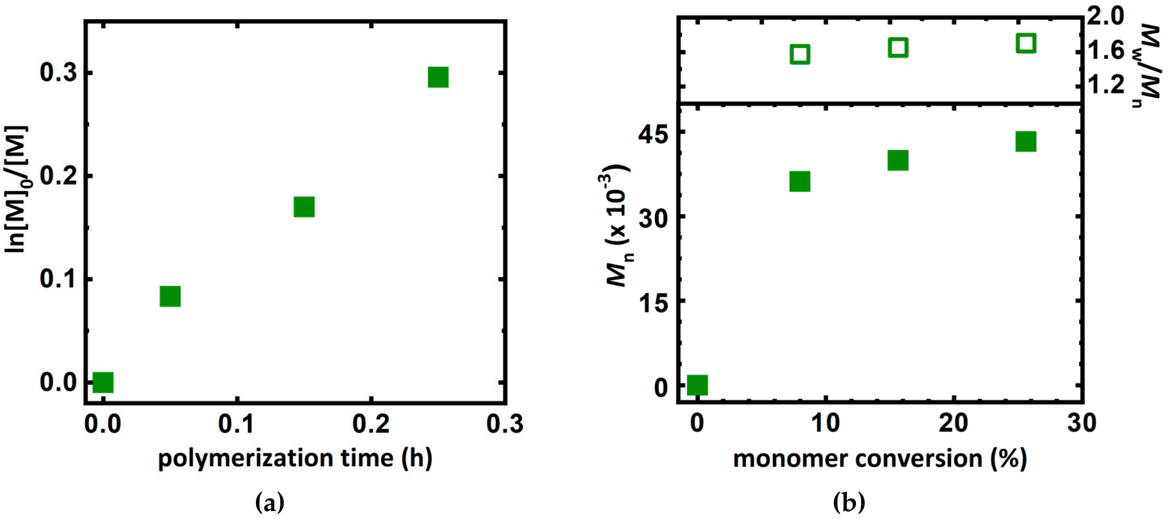

Figure S13. Polymerization of PDMAEMA (first block: Table S4 entry 1) in 7.5% coffee solution (a) First-order kinetic plot of monomer conversion *vs.* polymerization time. (b) M_n and M_w/M_n *vs.* monomer conversion.

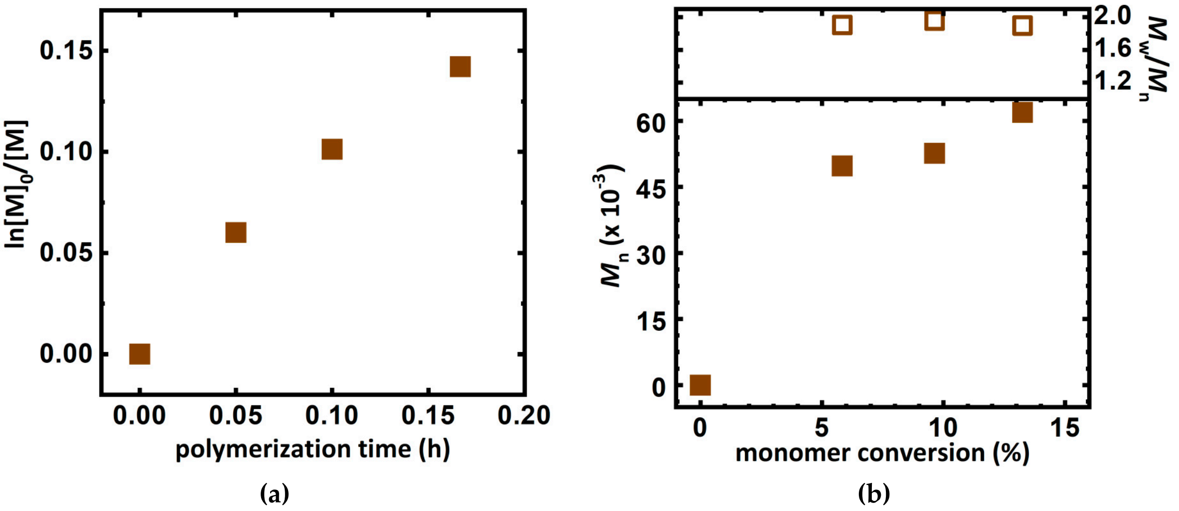

Figure S14. Copolymerization of PDMAEMA-Br (second block: Table S4 entry 2) in 7.5% coffee solution (a) First-order kinetic plot of monomer conversion *vs.* polymerization time. (b) M_n and M_w/M_n *vs.* monomer conversion.

S8. DPV analysis

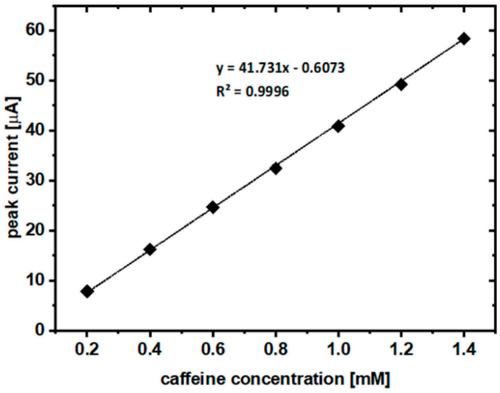

Figure S15. The calibration curve received for caffeine determination by DPV technique and the relative equation above the curve. The curve depicts linear dependance between the anodic peak current and caffeine concentration in 0.1 M H_2SO_4 aqueous solution. DP voltammograms received on GCE. DPV parameters: pulse potential of 50 mV, pulse time of 50 ms and scan rate of 50 mV/s.

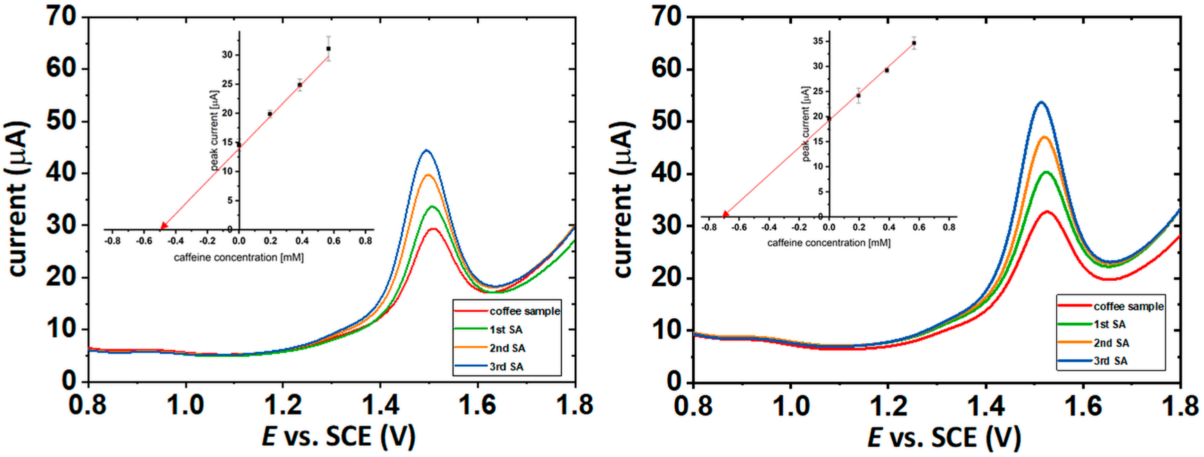

(a) (b)

Figure S16. DP voltammograms received on GCE in the analysis of coffee samples in 0.1 M H_2SO_4 aqueous solution using the standard addition method with 1 mL of the sample and after spiking 200, 400 and 600 μL of 10 mM caffeine standard solution, (**a**) 5% Arabica & Robusta (50/50%) sample, (**b**) 10% Arabica & Robusta (50/50%) sample. The analysis by standard addition method is presented in the insets. DPV parameters: pulse potential of 50 mV, pulse time of 50 ms and scan rate of 50 mV/s.

S9. HPLC analysis

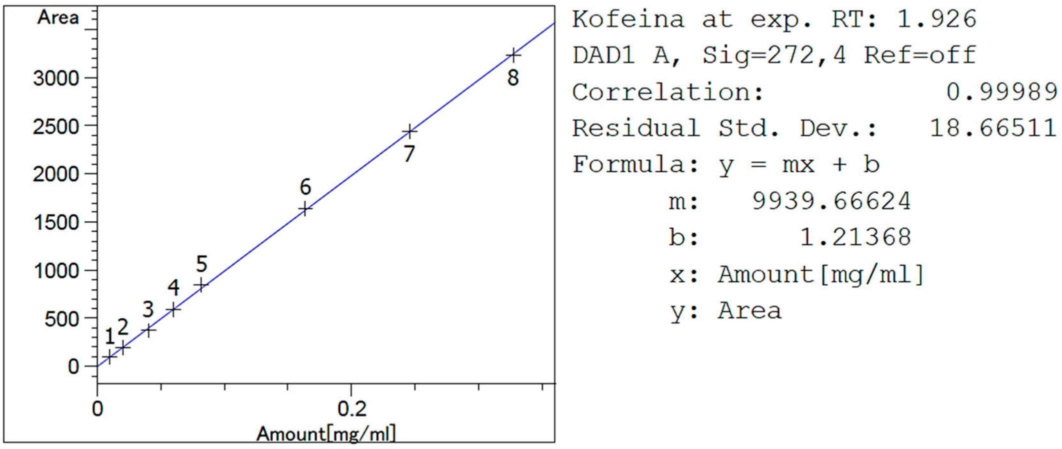

Figure S17. The calibration curve received for caffeine determination by HPLC technique and the relative equation on the side. Analysis performed with the use of 1290 Infinity LC system with DAD detector and Agilent ZORBAX Eclipse Plus C18, Rapid Resolution HT, 90A, (4.6 x 50 mm, 1.8 um, 600 bar) column in 30°C. Mobile phase: water (75%) and methanol (25%), flow rate: 1.0 ml/min. Wavelength used for caffeine detection: 272 nm.

S10. ARGET ATRP of DMAEMA in 7.0 mM caffeine solution

Table S5. ARGET ATRP of DMAEMA in 7.0 mM caffeine solution

**Entry**[1]	**t (h)**	**Conv**[2] **(%)**	$\mathbf{DP_{app}}$[3]	k_p^{app} [2] **(h^{-1})**	$M_{n,th}$[3] **($\times 10^{-3}$)**	$M_{n,app}$[4] **($\times 10^{-3}$)**	$Đ$ [4]
1	1.25	13.8	28	0.12	4.6	49.1	1.58

[1]General reaction conditions: $[DMAEMA]_0/[EBiB]_0/[Cu^{II}Br_2]_0/[TPMA]_0$: 200/1/0.01/0.02; T = 22°C; V_{tot} = 8 mL (DMAEMA/ 7.0 mM caffeine solution = 0.4/0.6 by v/v), $[DMAEMA]_0$ = 19 mM, $[I]_0$ = 0.09 mM. $[Cu^{II}Br_2]$= 1.19 μM (2-fold excess of TPMA), [NaBr] = 0.1 M.

[2] Monomer conversion, polymerization rate (k_p^{app}) and DP_{app} were determined by ^{1}H NMR.

[3] $M_{n,th} = ([M]_0/[I]_0) \times Conv \times M_{monomer} + M_{initiator}$.

[4] $M_{n,app}$ and $Đ$ was determined by DMF + 10 mM LiCl GPC with PS standards.

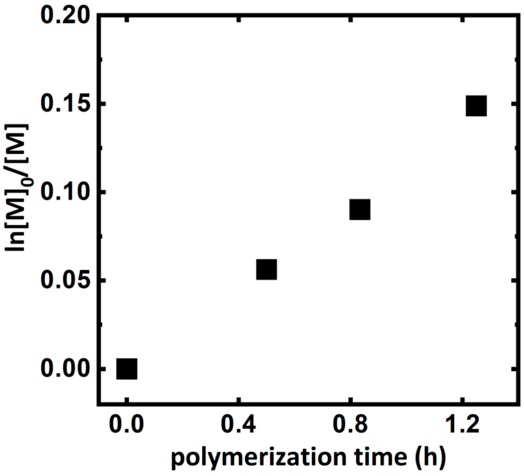

(a)

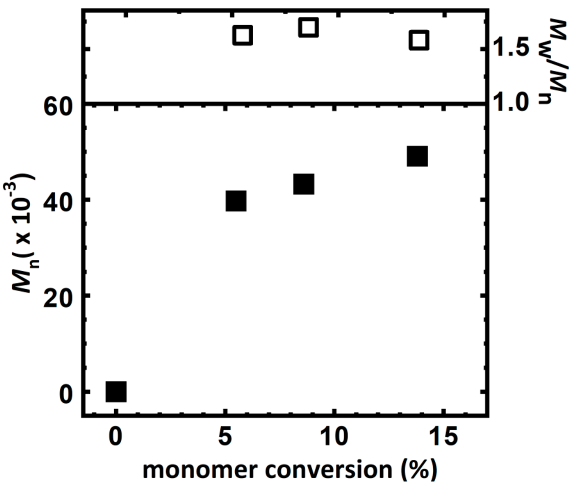

(b)

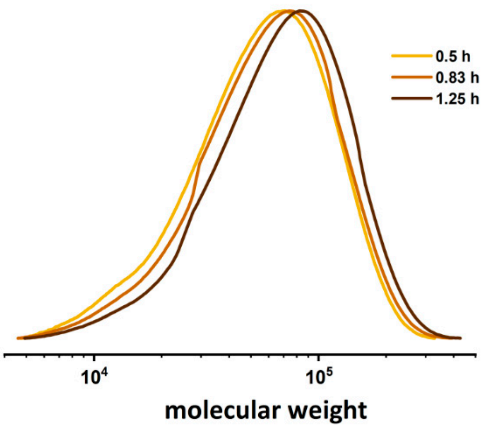

(c)

Figure S18. Polymerization of DMAEMA in 7.0 mM caffeine solution: (a) First-order kinetic plot of monomer conversion *vs.* polymerization time. (b) M_n and M_w/M_n *vs.* monomer conversion. (c) GPC traces of DMAEMA with DMF + 10 mM LiCl as the eluent.
